# Low-dose memantine-induced working memory improvement in the allothetic place avoidance alternation task (APAAT) in young adult male rats

**DOI:** 10.3389/fnbeh.2013.00203

**Published:** 2013-12-18

**Authors:** Malgorzata J. Wesierska, Weronika Duda, Colleen A. Dockery

**Affiliations:** ^1^Laboratory of Neuropsychology, Department of Neurophysiology, Nencki Institute of Experimental Biology, Polish Academy of SciencesWarsaw, Poland; ^2^Faculty of Life Sciences, Albstadt-Sigmaringen University of Applied SciencesSigmaringen, Germany

**Keywords:** working memory, cognitive skill learning, locomotor activity, MK-801, memantine, Allothetic Place Avoidance Alternation Task (APAAT)

## Abstract

N-methyl-D-aspartate receptors (NMDAR) are involved in neuronal plasticity. To assess their role simultaneously in spatial working memory and non-cognitive learning, we used NMDAR antagonists and the Allothetic Place Avoidance Alternation Task (APAAT). In this test rats should avoid entering a place where shocks were presented on a rotating arena which requires cognitive coordination for the segregation of stimuli. The experiment took place 30 min after intraperitoneal injection of memantine (5, 10, 20 mg/kg b.w.: MemL, MemM, MemH, respectively) and (+)MK-801 (0.1, 0.2, 0.3 mg/kg b.w.: MK-801L, MK-801M, MK-801H, respectively). Rats from the control group were intact or injected with saline (0.2 ml/kg). Over three consecutive days the rats underwent habituation, two avoidance training intervals with shocks, and a retrieval test. The shock sector was alternated daily. The after-effects of the agents were tested on Day 21. Rats treated with low dose memantine presented a longer maximum time avoided and fewer entrances than the MemH, MK-801M, MK-801H and Control rats. The shocks per entrances ratio, used as an index of cognitive skill learning, showed skill improvement after D1, except for rats treated by high doses of the agents. The activity levels, indicated by the distance walked, were higher for the groups treated with high doses of the agents. On D21 the MK801H rats performed the memory task better than the MemH rats, whereas the rats' activity depended on condition, not on the group factor. These results suggest that in naïve rats mild NMDAR blockade by low-dose memantine improves working memory related to a highly challenging task.

## Research highlights

Improvement of working memory was induced by low dose memantine;

The negative effects of high doses on cognitive function were diminished after a long break post-MK-801 administration, but not post-memantine.

The APAAT is a useful behavioral tool to study the effects of pharmacological treatments on both non-cognitive functions and the cognitive functions, learning, memory and executive functions.

## Introduction

Working memory is fundamental for sustaining successful daily activity in humans and animals. The brain areas involved in working memory include the prefrontal cortex, hippocampus and subcortical areas (Goldman-Rakic, 1995; Westerberg and Klingberg, 2007; Heuer and Bachevalier, 2012). The prelimbic and medial orbital cortices are part of the rat homologs of the human prefrontal cortex, which receive hippocampal projections from the ventral CA1 of the Ammon's horn and subiculum (Jay and Witter, 1991). A functional hippocampal-prefrontal network has been documented in the rat brain (Schwarz et al., 2013) and was found to be engaged in the rapid acquisition and short-term maintenance of spatial information (Burette et al., 2000). The prelimbic cortex is reciprocally connected with the rest of the prefrontal cortex (Jay and Witter, 1991); hence this area is capable of functionally integrating contributions from the hippocampus and prefrontal cortex, which further validate their participation in spatial working memory.

In human working memory function, temporary storage of current information occurs simultaneously along with the execution of higher cognitive function and skill performance (Baddeley, 1992; Repovs and Baddeley, 2006). It involves a multi-component system of short-term and long-term memory, which is distinguished by low and high capacities for information retention (Cowan, 2008). Animal models of working memory have utilized delayed alternation, the radial maze test, the water maze and the place avoidance test for their assessment (Dudchenko, 2004). The latter of these tasks, the place avoidance test with alternation of the target sector, involves a new set of spatial information about the task but does not require prior skill pretraining, which permits demonstration of skill improvement across time through training (Dockery and Wesierska, 2010).

It has been proven, that repetitive working memory training of a spatial task promotes improvement in the general learning performance and cognitive abilities (Klingberg, 2010). This improvement is known as cognitive skill learning (CSL), and just as for working memory in humans, it is domain-specific (e.g., visual-spatial or phonological information), and results in task-relevant improvements in terms of storage and assessment (Olesen et al., 2004; Lee et al., 2007). During CSL, such as evoked by exposure to novel task conditions, various learning mechanisms are involved including both declarative knowledge and procedural form (Anderson et al., 1997). CSL requires associative memory processes and intact fronto-striatal circuitry (Poldrack et al., 1999), in addition to normal activity in the dorsolateral prefrontal cortex (DLPFC) and the hippocampus (Cerella et al., 2006). Furthermore, there is a relationship between spatial working memory performance and motor skill learning, whereby spatial working memory, particularly in early learning, is predictive of the rate of motor learning in humans (specifically sensorimotor adaptation) with significant neural overlap between the two involving activation in the right DLPFC (Seidler et al., 2012).

CSL related to working memory in rodents has not, however, been shown to have an effect on the exploratory tendencies or other non-specific behavioral consequences of exposure to environments outside of the home cage (Light et al., 2010). Beyond the impact of training on CSL, improvement of working memory has been found as an immediate and latent effect of cathodal and anodal transcranial direct current brain stimulation (tDCS) when paired with training in humans (Dockery et al., 2009). The benefits of tDCS on spatial working memory and CSL in a rat model have furthermore been demonstrated in the place avoidance alternation task (Dockery et al., 2011). Interestingly, recent evidence suggests that anodal tDCS results in a reduction of GABA concentration, while cathodal stimulation decreases glutamate concentrations, in correlation with reduced GABA levels, as measured by magnetic resonance spectroscopy in the sensorimotor cortex (Stagg et al., 2009). It has been proposed that the cumulative benefits on working memory may result from homeostatic effects of tDCS through its interaction with mediators of neuronal function and plasticity in rats and humans (Dockery, 2013).

Activation of N-Methyl-D-aspartate (NMDA) receptors by glutamate is critical for long-term potentiation (LTP) and long term depression (LTD). They comprise a form of experience–dependent change in synaptic efficacy which is accepted as a cellular analog of learning, long-term memory storage (Lynch, 2004; Pastalkova et al., 2006), working memory and cognitive function (Timofeeva and Levin, 2011; Wang et al., 2013). Over expression of glutamate excitation involves increased intracellular Ca^2+^ and Na^+^ ions which generate excitotoxic effects. These effects are responsible for triggering neurodegeneration in many neurological diseases and disorders such as: AD, stroke, status epilepticus and head trauma. Impairment of working memory is a symptom of cognitive dysfunction which occurs with ageing, brain trauma, and neuropsychiatric diseases such as depression, Alzheimer's disease (AD) or schizophrenia (Elvevåg et al., 2000).

By studying the effect of NMDAR blockade on cognitive and non-cognitive processes, the role of NMDA receptors in the hippocampus, involved in working memory encoding and retrieval (Yoshihara and Ichitani, 2004), and their role in the prefrontal cortex, subserving persistent neuronal firing in the absence of sensory stimulation (Wang et al., 2013), can be further elucidated. Loss of the NMDA receptor NR1 subunit in the granule cells of the dentate gyrus impaired spatial memory (Niewoehner et al., 2007), and NMDA receptor deletion has been shown to both restrict CA3 pyramidal cells (Nakazawa et al., 2003) and affect spatial working memory. Moreover, blockade of NMDARs in the different subregions of the hippocampus has been found to affect different stages of spatial working memory (Lee and Kesner, 2002).

The NMDAR is a complex comprised of several heterogeneous subunits, which contains binding sites for the different modes of action of non-competitive antagonists such as (+)MK-801 (commercial name: Dizocilpine) or memantine (Paoletti et al., 2013). MK-801 acts without subunit selectivity and, with a long dwell-time in the ion channel, results in a slow off-rate and high affinity to NMDAR (Wong et al., 1986; Chen and Lipton, 2006). Memantine acts via a shorter dwell-time (faster off-rate), lower affinity and higher voltage dependence (Parsons et al., 1995; Chen and Lipton, 2006). It blocks excessive NMDA receptor activity without impairing normal activity, which is a feature of uncompetitive antagonists. Blockade of NMDA receptors by memantine and MK-801 has been proposed as a therapeutic intervention for neuroprotection in neurodegenerative diseases such as Alzheimer and Parkinson's Disease or stroke (Zajaczkowski et al., 1996; Danysz et al., 1997). However, the different properties of the two agents in regard to NMDARs influence the differential effects on cognitive and non-cognitive behavior.

NMDAR activity relates to normal and abnormal function of the nervous system via their excitatory activity (Chen and Lipton, 2006), whereby the levels of transmission represent a continuum with polarities between excessive and inadequate transmission. The critical aspect then in achieving therapeutic efficacy to ameliorate neurological diseases and psychiatric disorders, is through the capacity to register the current state of the NMDAR activity on this continuum and apply appropriate dosing of NMDAR antagonism to achieve health and reinstate proper function. MK-801 application in animal studies was found to impair food consumption, and disturb mobility (locomotor activity) and psychogenic activity (stereotypic activity and ataxia) (Mondadori et al., 1989; Whishaw and Auer, 1989), in addition to psychotic dysfunction in rats (Manahan-Vaughan et al., 2008). In healthy humans, MK-801 administration has been associated with hallucinations, delusions and affective blunting. Memantine in high doses has also been found to disturb motor function and spontaneous responses such as rearing (Creeley et al., 2006). However, in contrast to MK-801, for memantine the distinction between doses which produced undesirable side effects and those which elicit promising therapeutic effects are clearly distinguishable (Morè et al., 2008). A pharmacological study showed that injection of 5 mg/kg of memantine resulted in plasma concentrations in a therapeutic range (about 1.0 umol/l), which is safe and does not cause learning and memory impairment. Whereas higher doses of memantine (10 mg/kg) were found to produce plasma level concentrations that were fivefold higher than those which mediated therapeutic effects in patients requiring treatment (Zoladz et al., 2006).

The effects of MK-801 and memantine have been studied in relation to cognitive function in animal models of long- and short-term memory, but have rarely been tested in working memory paradigms. The effects on memory have been shown to be dose- and time-dependent. When applied 30 min before the experiment both memantine (in 0.5 or 1.0 mg/kg doses) and MK-801 (in 0.025 or 0.05 mg/kg doses) preserved intact working memory performance in a delayed match–to–position task (Smith et al., 2011). Whereas memantine in 5 mg/kg or MK-801 in801 in 0.1 mg/kg doses impaired working memory in the same test. The low and high doses of MK-801 (0.25, 0.5, 1.0, 4.0 mg/kg) made working memory worse in the water maze test shortly after injections (from 3h to 1 day post-treatment), but not on the third or the fourth post-treatment day (Whishaw and Auer, 1989). In the radial maze, working memory was intact in rats with high doses of memantine (20 mg/kg per day) and MK-801 (0.312 mg/kg per day) applied as a chronic infusion during the experiment (Zajaczkowski et al., 1996) or post-administration of a 5 mg/kg dose of MK-801 and high doses of memantine (20 and 40 mg/kg) with a long delay (8 days) (Zajaczkowski et al., 2000). Also acute application of moderate doses of memantine (2.5, 5.0, 10.0 mg/kg) preserved short-term memory in the radial water maze test (Zoladz et al., 2006). After a high dose of memantine (20 mg/kg) a slight improvement in working memory was observed at the onset of training in the radial maze (Zajaczkowski et al., 2000).

The experimental results collectively suggest palliative effects of memantine in low doses with mixed results for MK-801. As a consequence of such experimental results, memantine received authorization as a treatment for AD in 2002 in the EU and in 2003 in the USA (Parsons et al., 2007). In contrast, MK-801 dose-dependently induced mobility disturbances and long-lasting spatial memory impairment (Zajaczkowski et al., 1996), which mimics symptoms of schizophrenia. Therefore, application of MK-801 has been proposed as an animal model of schizophrenia using the active allothetic place avoidance method (Vales et al., 2010).

In active place avoidance tasks, a freely walking rat must remember and avoid an unmarked place on an elevated arena where foot-shocks are administered. When associated with a rotating arena in light, this place, or “to-be-avoided sector,” is oriented according to room frame coordinates in which place avoidance demands segregation of relevant extramaze room stimuli from irrelevant intramaze arena stimuli. To achieve accurate memory performance in the active place avoidance task, segregation of relevant from irrelevant arena frame stimuli is required, a process which engages cognitive coordination (Wesierska et al., 2005). The inability to segregate stimuli has been proposed as a disturbance in cognitive coordination which is found in patients with schizophrenia (Phillips and Silverstein, 2003).

In order to specifically test working memory and CSL, a variant of the active place avoidance, the Allothetic Place Avoidance Alternation Task (APAAT), has been developed (Dockery and Wesierska, 2010). Due to the alternation aspect of the APAAT task, in which the to-be-avoided sector is alternated daily, new information is needed for each session in order for rats to perform place avoidance correctly. As training progresses, simultaneously the memory load also increases, as rats are obliged to continually update the location of the to be-avoided sector since its location changes for each day. To date a dose-response relationship concerning the effects of NMDAR blockade on APAAT performance has not been established. By determining the dose-response curve in this task, the possible remedial effects of the relevant drugs could be clarified. The aim of the presented study was to compare the cognitive and non-cognitive processes during, immediately after, and following a long delay post-drug administration of a range of doses of memantine and MK-801 in this task. Thereby, the APAAT allowed for simultaneous examination of spatial working memory capacity and the efficiency of CSL, in addition to locomotor activity as a non-cognitive process. Such comparisons have never been previously conducted.

## Materials and methods

### Animals

Eighty-one naïve adult (3.5-month-old) male Long Evans rats, weighing 270–360 g, were obtained from the breeding colony of the Nencki Institute of Experimental Biology, Polish Academy of Science, Warsaw, Poland. They were accommodated in transparent plastic home cages, four per cage, under standard conditions (a constant temperature of 22°C, 12:12 light/dark cycle, humidity at 23%). Water and food were available in the cages *ad-libitum*. The animals were handled for four days prior to the onset of the experiment. All manipulations were done according to the European Community Directive for the ethical use of experimental animals and the Polish Communities Council for the care and use of laboratory animals.

### Drug treatment

Memantine—(3, 5-Dimethyl-1-adamantanamine hydrochloride, 3,5-Dimethylamantadine hydrochloride; Sigma Aldrich) was dissolved in saline (5, 10, 20 mg/ml) and injected intraperitoneally (5, 10, 20 mg/kg b.w.). (+)MK-801–((5S,10R)-(+)-5-Methyl-10,11-dihydro-5H-dibenzo[a,d]cyclohepten-5,10-imine hydrogen maleate, Dizocilpine hydrogen maleate; Sigma Aldrich) was dissolved in saline solution (0.1, 0.2, 0.3 mg/ml) and injected intraperitoneally (0.1, 0.2, 0.3 mg/kg b.w.). For both drug treatments, the animals received the same volume of liquid per kg of body weight which was applied 30 min before the training sessions on Days 1, 2, and 3.

### Apparatus

The active allothetic place avoidance apparatus was previously described in detail (Wesierska et al., 2009). Briefly, the apparatus consisted of an 80-cm-diameter, rotating (1 rpm) platform or “arena” made of aluminum. It was elevated (80 cm), and located in a room with dim light and explicit visual landmarks (pictures, lamp, furniture). Rats wore a latex harness on their back upon which an infrared light-emitting diode (LED) was fixed. The second LED was attached to the periphery of the arena. The infrared TV camera was connected to a computer system which allowed for monitoring the position of the rat. Rats were pierced between the shoulders with a subcutaneous connector (surgical needle) which provided an anchor for a mini-alligator clip that was connected by a cable to the shock box. Every time the rat entered the to-be-avoided sector (60°) a mild, constant current foot-shock was delivered through the connector placed on the rat's back. The shock was repeated every 1.5 s until the rat escaped from the shock sector. The data were collected and analyzed by the place avoidance system (Bio-Signal Group, Brooklyn, New York).

### Experimental groups

Rats (*n* = 81) were randomly divided into eight groups. The Control group of rats consisted of intact animals (*n* = 12) and rats treated with saline (*n* = 11; 1 ml/kg b.w.). The memantine group was divided into three subgroups according to the dosage: a low (MemL) 5 mg/kg (*n* = 12), medium (MemM) 10 mg/kg (*n* = 12) and high (MemH) 20 mg/kg (*n* = 10) dosage group. Likewise, the dizocilpine ((+)-MK-801) group of rats was divided into three subgroups: a low (MK-801L) 0.1 mg/kg (*n* = 8), medium (MK-801M) 0.2 mg/kg (*n* = 8) and high (MK-801H) 0.3 mg/kg (*n* = 8) dosage group (Table 1).

**Table 1:**
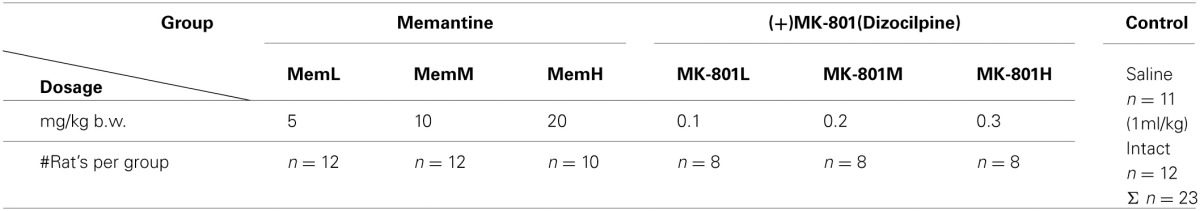
**Description of experimental groups**.

### Behavioral procedures

The experiment was divided into two stages. The first stage consisted of Days 1, 2, and 3 (D1, D2, D3), during which rats were injected with drugs and underwent training and testing sessions on the rotating arena. The second stage consisted of a single session, Day 21 (D21), which served as a follow-up to test the long-term influence of prior memantine and MK-801 injections paired with training on performance in the APAAT. On D21 rats were not injected before the behavioral session, however, the behavioral conditions were the same as for the first stage.

The experiment started on Day 0 which served as the habitation during which the rats were placed on the rotating arena for 5 min without an active shock sector. The next days (D1, D2, and D3) were training days during which the rats were injected intraperitoneally 30 min before the avoidance training began. Each behavioral session began with habituation (ha) on the rotating arena with an inactive shock sector. After 5 min of habituation the to-be-avoided sector was activated. The rats which were able to avoid the sector consistently for at least 90 s. completed the two training intervals after 10 min (5 min for tr1 and 5 min for tr2) and were removed from the arena. If a rat did not reach this criterion before the end of the 10 min, they underwent an additional 5 min of training. Those rats then underwent a 5 min delay period in a cage in an adjacent room. Afterwards, they were returned to the rotating arena for the 5 min retrieval test (ts). During the test the original shock sector was inactivated. The to-be-avoided sector was defined by room frame coordinates and was changed each day according to the following order: D1–Northwest, D2–Northeast, D3–Southwest, D21–Southeast.

### Measures

The independent variables taken to describe cognitive working memory processes included the number of entrances into the shock sector (ENTR), the maximum time spent avoiding the shock sector (s) (Tmax) and the number of shocks per entrance (SH/ENTR). For habituation and the retrieval test, the shock was inactivated, however, the program still registered undelivered number of shocks related to the rat's presence in the to-be-avoided sector. This provided an index of “untrained behavior.” During training, a high SH/ENTR ratio expresses poor CSL. Non-cognitive functions were assessed by locomotor activity by the total path length (distance) (m) and linearity (Lin). The latter was calculated as the average ratio of linear distance during each two-second time bin divided by the sum of the path distance determined by each 20 ms epoch.

### Data analysis

Based on the above measures for D1–3, analysis of the across session effects on performance was performed by a three-way ANOVA (group (MemL, MemM, MemH, MK-801L, MK-801M, MK-801H, CONTR) × day (D1, D2, D3) × condition (ha, tr1, tr2, ts): 7 × 3 × 4; with repeated measures on the last factors) followed by a Tukey HSD multiple comparison *post-hoc* test. On D21, a two-way ANOVA (group (MemL, MemM, MemH, MK-801L, MK-801M, MK-801H, CONTR) x condition (ha, tr1, tr2, ts): 7 × 4; with repeated measures on the last two factors) was performed with a Tukey HSD test for multiple comparisons. Significance was accepted at a level of *P* < 0.05. The statistical analysis was performed with STATISTICA 7.1. Group averages and ±s.e.m. values are reported.

## Results

### Dose-related effects of memantine and MK-801 administration on working memory training (days 1–3) evaluated by the number of entrances into and maximum time spent avoiding the shock sector. high doses of both agents impaired working memory, while low doses helped maintain or even improve (memantine) it

**Figure 1:**
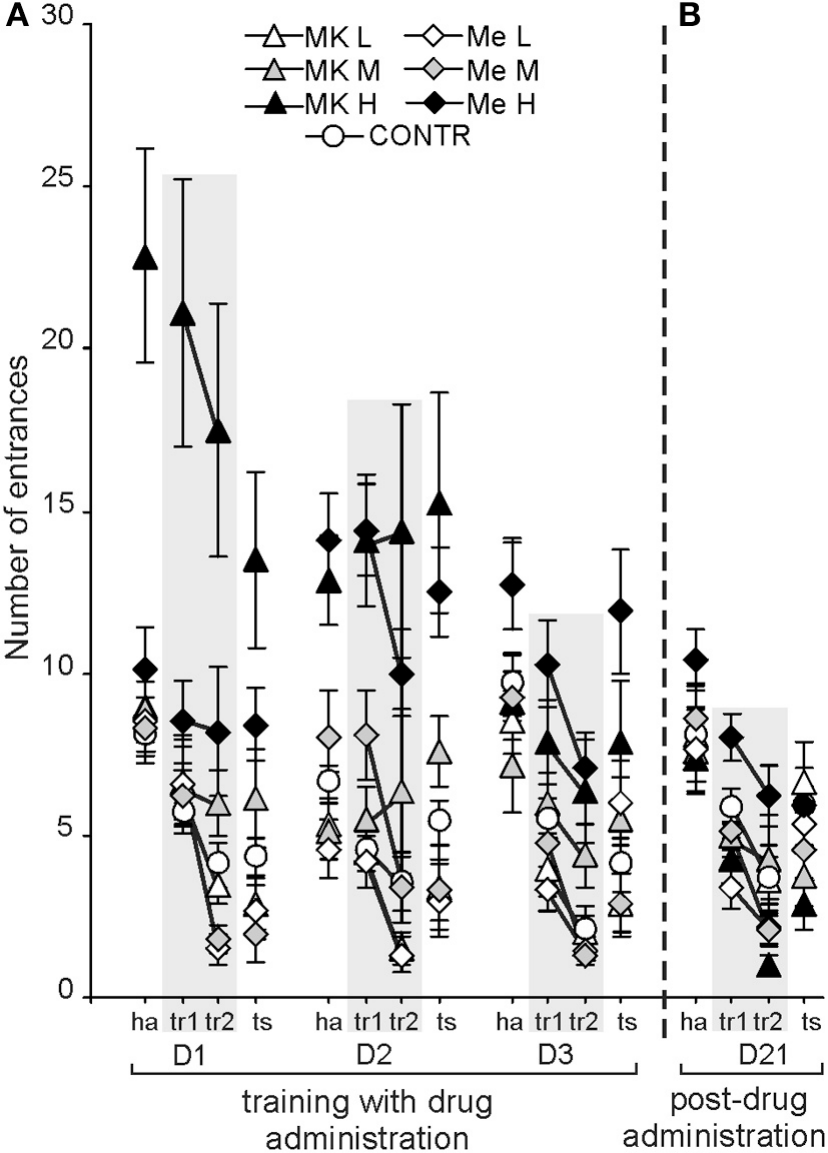
**The high dose of memantine and MK-801 (MemH and MK-801H, respectively), in contrast to the low doses, impaired working memory performance in the allothetic place avoidance alternation task as shown by a high number of entrances for D1–3 (A).** This effect was absent for the long-term follow-up when no drug was administered (D21), however, rats previously treated with MemH performed worse than those previously treated with MK-801H **(B)**. The values are presented as the average number of entrances (±s.e.m.) for groups, across all days and for all conditions. The values are presented as the grand average (±s.e.m.) according to group, day and condition. Memantine doses: low MemL–5 mg/kg, medium MemM–10 mg/kg and high MemH–20 mg/kg. (+)MK-801 doses: low MK-801L–0.1 mg/kg, medium MK-801M–0.2 mg/kg and high MK-801H–0.3 mg/kg. The shaded columns represent the conditions during which shock was activated.

Working memory performance expressed as the number of entrances (ENTR) into the shock sector changed according to the agents used and their doses [*F*_(6, 74)_ = 29.068; *P* < 1× 10^−16^; MemH, MK-801H > CONTR, MK-801L, MK-801M, MemL, MemM], across days [*F*_(2, 148)_ = 5.42; *P* < 0.005; D1, D2 > D3] and for the different conditions within a session [*F*_(3, 222)_ = 74.70; *P* < 0.000001; tr2 < ts < tr1 < ha] (Figure 1A). Working memory was worse (more entrances) after the high doses of both agents (*P* < 0.0001). Rats performed better on D3 than on D1, and D2 (*P* < 0.001). Although during the test condition rats had more entrances than during tr2, it was significantly less than in tr1 and ha (*P* < 0.0008). The *post-hoc* evaluation of the group by day interaction [*F*_(12, 148)_ = 6.27; *P* < 6.7 × 10^−8^] confirmed that rats from the MemL, MK-801L, and CONTR groups showed a lower number of ENTR across the consecutive days. Their performance was better (fewer entrances) than in the rats with MemH on D2 and D3, and MK-801H on D1 and D2 (*P* < 0.001). The *post-hoc* test for the group by condition interaction [*F*_(18, 222)_ = 3.26; *P* < 1.8 × 10 ^−4^] showed that avoidance during tr2 was better than in the other conditions for the MemL, MK-801L, and CONTR groups (*P* < 0.01). In contrast, the number of ENTR for MemH and MK-801H was at a similar level for all the session conditions and was worse (more entrances) than in the other groups (*P* < 0.001). On D3 the number of ENTR during tr2 was lower than for the other session conditions for all days as shown by a *post-hoc* for the day by condition interaction [*F*_(6, 444)_ = 10.69; *P* < 4 × 10^−10^; *post-hoc* test; *P* < 0.0004].

**Figure 2:**
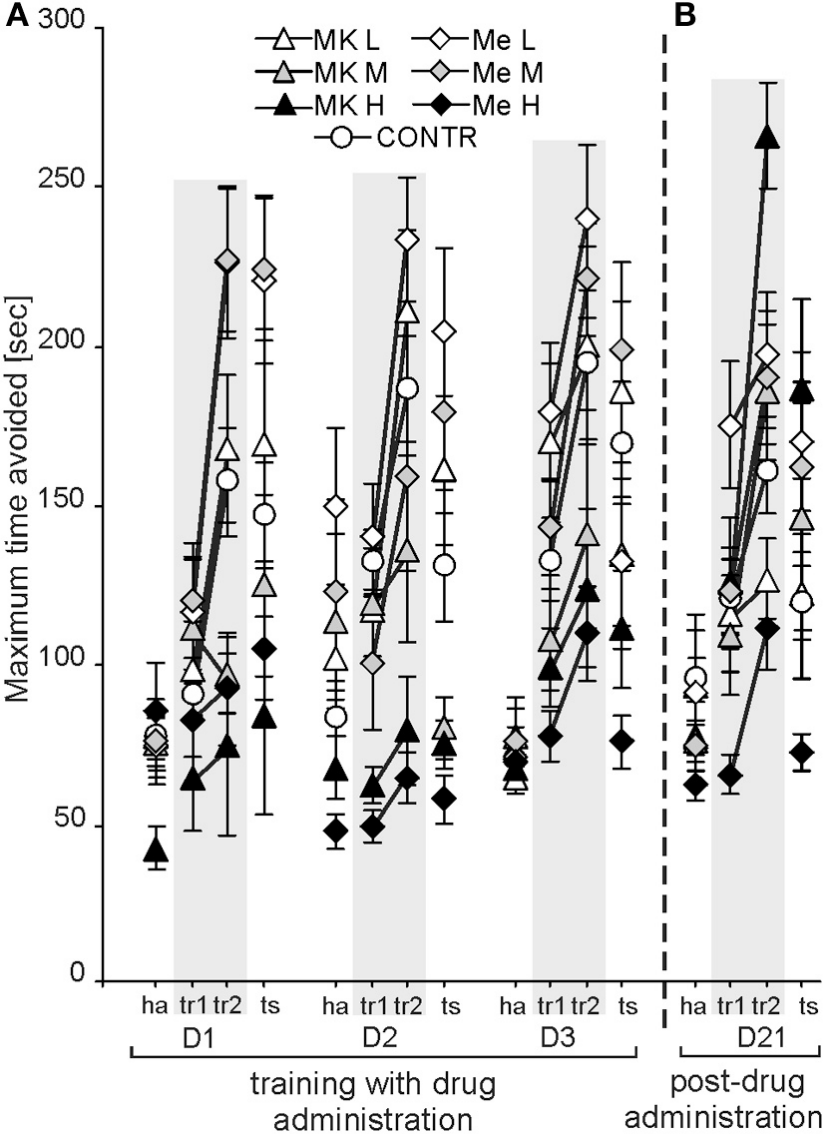
**The benefit of low-dose memantine on working memory performance was evidenced by a longer maximum time avoided (Tmax) in comparison to the control, MemH and MK-801H rats (*P* < 0.02; *P* < 0.0001; *P* < 0.0001, respectively) **(A)**.** Without any drug administration (D21) working memory performance was similar for all groups, but post-treatment MemH rats were worse than post-MK-801H **(B)**. The values are presented as the average Tmax (±s.e.m.) for groups across days and for all conditions. Other figure elements are the same as in Figure 1.

In the APAAT, the maximum time avoided (Tmax) is used to represent working memory performance. It differed depending on the group [*F*_(6, 74)_ = 18.70; *P* < 4 × 10^−12^; MemL > CONTR, MemH,MK-801H, MK-801M] and condition [*F*_(3, 222)_ = 83.39; *P* < 0.00000; tr2 > ts > tr1 > ha], whereas there was no significant effect of days (Figure 2A). The *post-hoc* test confirmed that rats treated with MemL avoided better (a higher Tmax) than the CONTR (*P* < 0.02), MemH, MK-801M, and MK-801H (*P* < 0.001; *P* < 0.0001) groups. No differences were found between the MemL and MK-801L, or between the MK-801L and control rats. The rats treated with MemH or MK-801H performed worse than the rats from the other groups. For condition, the shock sector was avoided with a longer Tmax during tr2 than during the other conditions (*post-hoc*; *P* < 0.002). Although the Tmax during the retrieval test (shock inactivated) was shorter than during tr2 (*post-hoc* test; *P* < 0.0008), it was longer than the Tmax during tr1 and ha (*post-hoc* test; *P* < 7 × 10^−6^). The *post-hoc* for the group by condition interaction [*F*_(18, 222)_ = 4.86; *P* < 3 × 10^−8^] showed that the Tmax during tr2 was shorter for the control than for MemL (*P* < 0.02), MemH and MK-801H (*P* < 0.0001) groups, and similar as that for the MK-801L, MK-801M, and MemM groups. The rats treated by high memantine or MK-801 presented a low Tmax for all conditions. The *post-hoc* for the day by condition interaction [*F*_(6, 444)_ = 5.59; *P* < 1 × 10^−4^; *P* < 0.02] showed that the Tmax was similar for tr2 across all days. The Tmax was higher for tr2 than for tr1, ha (D1-3) and ts (except for on D1).

### Dose-related effects of memantine and MK-801 administration on cognitive skill learning (days 1–3) evaluated as the shocks per entrance ratio (SH/ENTR). rapid acquisition of cognitive skill learning (within the first session) occurred and was maintained over the long-term (between sessions) after treatment with low dosages of both of the NMDAR antagonists

**Figure 3:**
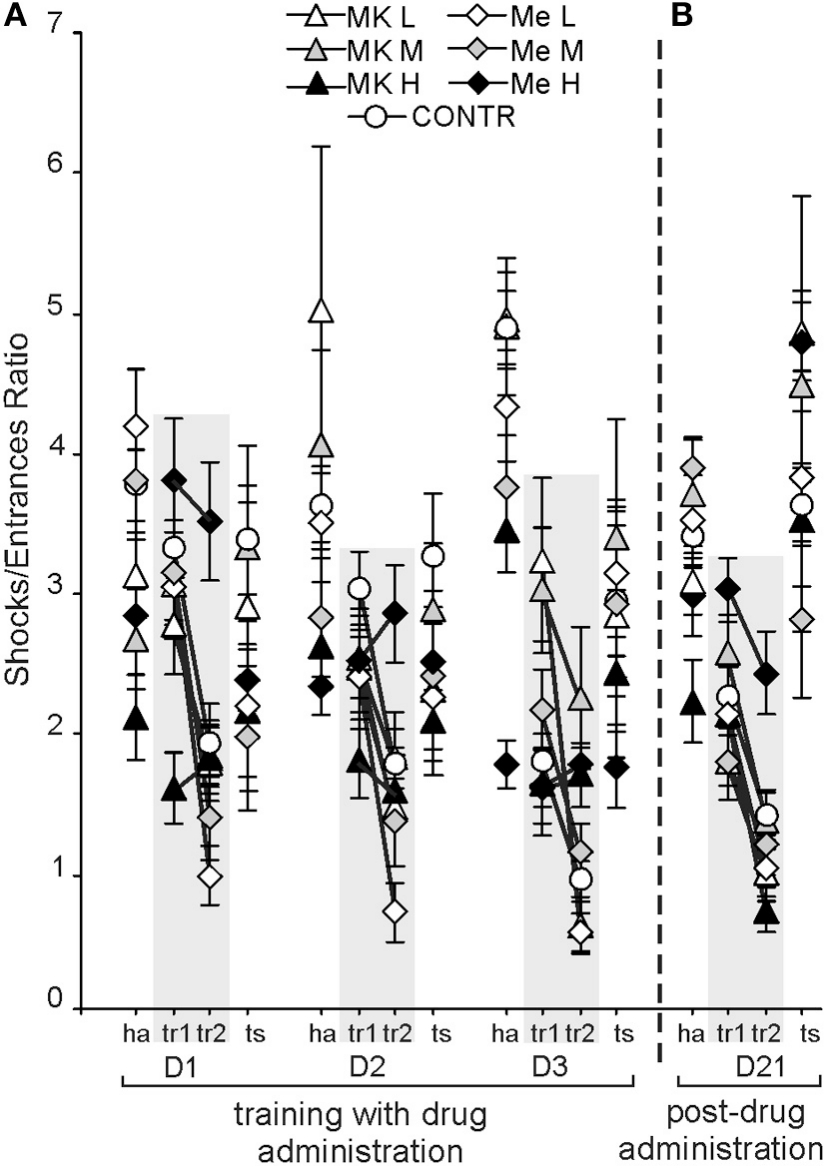
**Cognitive skill learning in the allothetic place avoidance alternation task was calculated as the ratio of shocks per entrances (SH/ENTR) into the shock sector which developed according to day and condition **(A)**.** On D1 the ratios for ha and tr1 were similar, whereas for D2 and D3 it decreased for tr1 in comparison to ha. Without any drug administration (D21), all rats presented similar skill levels in which performance was dependent on condition **(B)**. The values are presented as the grand average (±s.e.m.) according to group, day and condition. Other figure elements are the same as in Figure 1.

CSL was expressed as the shock per entrances ratio (SH/ENTR). It was dependent on condition [*F*_(3, 222)_ = 77.48; *P* < 0.0000)(ha > tr1, ts > tr2], but not day [*F*_(2, 148)_ = 0.83; *P* = 0.43] (Figure 3A). Although the main effect of treatment [*F*_(6, 74)_ = 2.91; *P* < 0.013] was significant, the *post-hoc* did not show a significant difference between groups. The SH/ENTR ratio was the highest during habituation (*P* < 6 × 10^−5^) and the lowest during tr2 (*P* < 7 × 10^−4^). The *post-hoc* test for the group by day interaction [*F*_(12, 148)_ = 2.22; *P* < 0.013] showed that the SH/ENTR ratio was similar for all groups on D1 and D2. On D3 the ratio for MemH was lower than that for MK-801M only (*P* < 0.001).

The *post-hoc* evaluation of the group by condition interaction [*F*_(18, 222)_ = 5.35; *P* < 2 × 10^−9^; *P* < 0.01] confirmed that during tr1 the SH/ENTR ratio was lower than during ha for the CONTR, MK-801L and MemL groups (*P* < 0.01), whereas rats treated with middle or high doses of MK-801 and memantine presented similar ratios during ha and tr1. Rats from the high MK-801 and memantine groups presented similar ratios across all conditions. The *post-hoc* evaluation for the day by condition interaction [*F*_(6, 444)_ = 6.27; *P* < 2 × 10^−5^] showed that the ratios for ha and tr1 on D1 were similar, whereas on the next days the ratio for tr1 decreased compared to ha (*P* < 0.0003).

### Dose-related effects of memantine and MK-801 administration on non-cognitive functions expressed by the distance walked by rats and the linearity (straightness), of the rat's path. the high dose of MK-801 was associated with hyperactivity, but did not affect linearity

**Figure 4:**
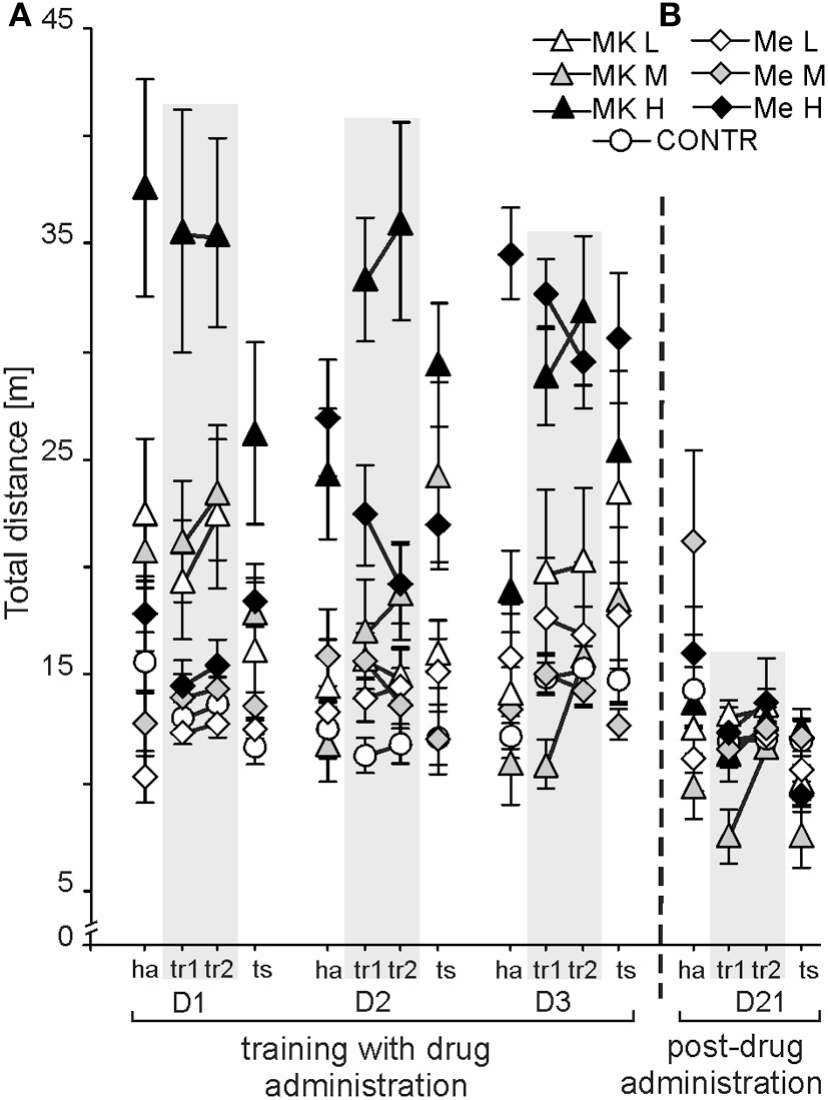
**Non-cognitive function was represented by the activity levels measured by the distance [m] walked in the allothetic place avoidance alternation task.** After the high dose of MK-801 (MK-801H) the activity level was high across all days (D1-3), while it was only high for D3 following the high memantine dose (MemH) **(A)**. Without any drug administration (Day 21), hyperlocomotion in MK-801H rats post-treatment ceased and locomotion returned to a similar level as in other post-treated rats **(B)**. The values are presented as the grand average (±s.e.m.) according to group, day and condition. Other figure elements are the same as in Figure 1.

The distance walked by rats was dependent on treatment and condition as confirmed by a significant effect of group [*F*_(6, 72)_ = 21.01; *P* < 4 × 10^−13^; MK-801H > all other groups] and condition [*F*_(3, 216)_ = 4.69; *P* < 0.003; tr2 > ha, tr1, ts], whereas an effect of day was not significant [*F*_(2, 144)_ = 2.34; *P* = 0.099] (Figure 4A). The *post-hoc* test for groups showed that the MK-801H rats walked more than the other rats (*P* < 0.0001; *P* < 0.05 for MemH). The rats from the CONTR group walked a shorter distance than the rats treated by high doses of memantine and MK-801. The *post-hoc* test for condition confirmed a longer distance for tr2 than for the other conditions (*P* < 0.004). Rats presented a similar distance during tr1, ha, and ts. The *post-hoc* evaluation of the group by day interaction [*F*_(12, 144)_ = 8.81; *P* < 1.7 × 10^−11^] confirmed that the MK-801H rats walked a similarly long distance every day, which was similar to that of the MemH rats on D3 (*P* < 1.7 × 10^−5^). Rats treated with MK-801H walked a long distance for all conditions [group by condition interaction *F*_(18, 216)_ = 5.08; *P* < 1.2 × 10 ^−8^; *P* < 0.001]. The *post-hoc* evaluation of the day by condition interaction [*F*_(6, 432)_ = 7.19; *P* < 2.5 × 10 ^−6^] confirmed a shorter path during habituation on D2 and D3 than for the other conditions (*P* < 0.01). No differences were found between tr1 and tr2 for all days.

**Figure 5:**
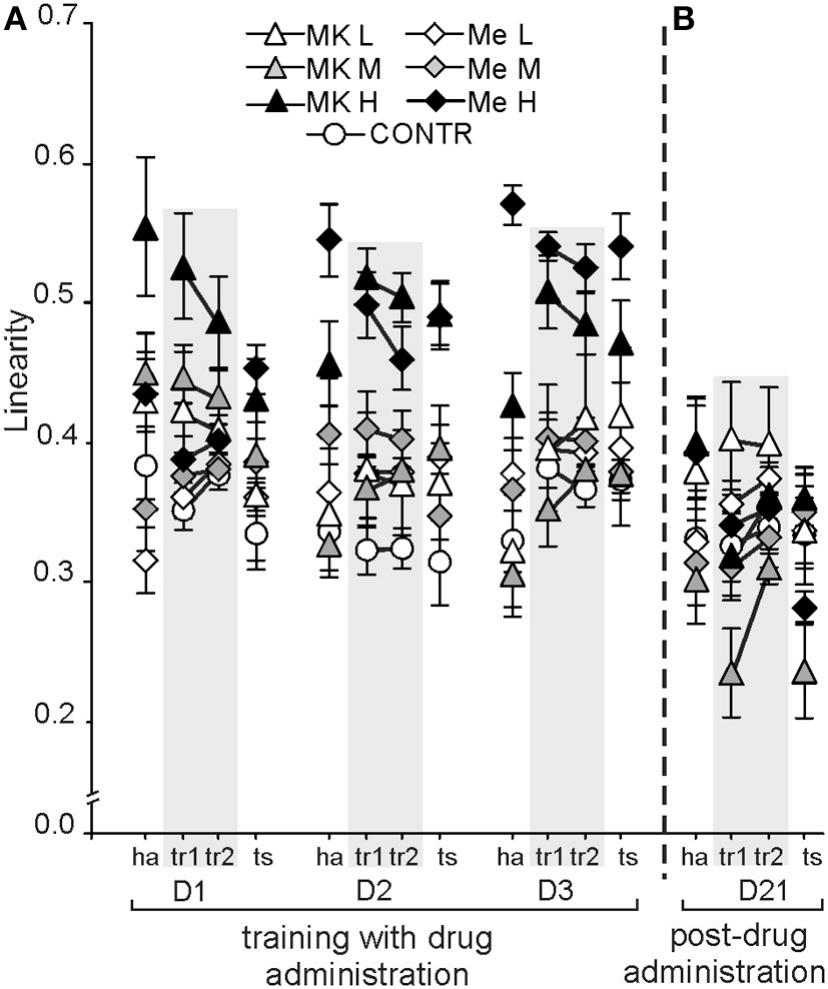
**The linearity of the path on the arena in the allothetic place avoidance task was straighter for the MK-801H rats across days (D1–3), and on D2 and D3 for the MemH rats (A).** On D21, the linearity was dependent on condition **(B)**. The values are presented as the grand average (±s.e.m.) according to group, day and condition for rats treated with different doses of memantine or MK-801. Other figure elements are the same as in Figure 1.

Linearity, which represents the straightness of a rat's path in the arena according to a 20 ms sampling rate, changed dependent on treatment [*F*_(6, 72)_ = 16.22; *P* < 10 × 10^−10^; Control, low and MK-801M and MemM < MK-801H and MemH] and condition [*F*_(3, 216)_ = 2.78; *P* < 0.04; tr1 > ha] (Figure 5A). Rats from the high MK-801 and memantine groups walked straighter than the other rats (a high linearity value) (*P* < 0.002). Rats walked straighter during tr1 than during ha and ts (*P* < 0.03), with no differences found between tr2 and the other conditions. The *post-hoc* evaluation of the group by day interaction [*F*_(12, 144)_ = 5.54; *P* < 9 × 10^−7^] showed that MK-801H rats presented a similar linearity value across days, which was similar to MemH except for on D1, but was higher than in the other groups of rats (*post-hoc* test; *P* < 0.001). The *post-hoc* evaluation of the group by condition interaction [*F*_(18, 216)_ = 1.91; *P* < 0.016] confirmed that the MK-801H and MemH rats had a similar linearity across conditions, which was higher than in the other groups independent of condition (*P* < 0.0001). The linearity values during ha and tr1on D1 and D2 were similar, which were lower than during tr1, tr2, and ts on D3 (*post-hoc*; *P* < 0.01) for the day by condition interaction [*F*_(6, 432)_ = 4.941; *P* < 6 × 10^−4^].

### Long-term effects (D21) on working memory without drug administration

Proper functioning of working memory post-drug administration and early training (D21) was evidenced by a low number of entrances with a long maximum time avoided and via demonstration of the effective skill to avoid the new to-be-avoided sector.

Working memory performance in control rats and rats after memantine or MK-801 administration was assessed on D21 according to the number of entrances. A significant effect was confirmed for group [*F*_(6, 74)_ = 3.98; *P* < 0.002] and condition [*F*_(3, 222)_ = 34.501; *P* < 2 × 10^−18^] (Figure 1B). The *post-hoc* for group confirmed that MK-801H rats (no drug administered) performed better than MemH rats (*P* < 0.002), but the difference between the control and other treatment groups was not significant. The number of ENTR during tr2 was lower than during the other conditions (ha > tr2 < tr1, ts; *P* < 0.001).

The maximum time avoided showed significant effects of group [*F*_(6, 74)_ = 6.37; *P* < 1, 9 × 10^−4^] and condition [*F*_(3, 222)_ = 37.47; *P* < 1, 2 × 10^−18^], and a group by condition interaction [*F*_(18, 222)_ = 1.80; *P* < 0.03] (Figure 2B). Although the MK-801H rats presented a longer maximum avoidance time than the MK-801L, MK-801M and MemH rats (*P* < 0.04), it was similar to that for the control rats. The Tmax was longer during tr2 than during the tr1, ts and ha conditions (*P* < 0.0001), but no difference was found between tr1 and ts. The *post-hoc* test for the group by condition interaction confirmed that during tr2 the rats from the MK-801H group had a longer Tmax than the rats from the MK-801L, MemH and CONTR groups (*P* < 0.03). The MemH rats presented a shorter Tmax across all conditions.

The SH/ENTR ratio on D21 showed significant effects for condition [*F*_(3, 222)_ = 74.04; *P* < 0.00001] and for the group by condition interaction [*F*_(18, 222)_ = 2.06; *P* < 0.008]. There was a low ratio during tr2 and a high ratio during the ts compared to during tr1 and ha (*post-hoc* test; *P* < 0.0002). The ratio during tr1 was lower than during ha and the ts (*post-hoc* test; *P* < 1 × 10^−7^). The *post-hoc* test for the interaction showed similar SH/ENTR values during tr2 across groups (Figure 3B).

### Long-term effects (D21) on non-cognitive function without drug administration

On D21 the locomotor activity was dependent on condition [*F*_(3, 219)_ = 9.66; *P* < 5 × 10^−5^] and showed a group by condition interaction [*F*_(18, 219)_ = 2.41; *P* < 0.0015] (Figure 4B). All rats walked more during habituation and tr2 than during tr1 and ts (*P* < 0.0001). The *post-hoc* for the group by condition interaction confirmed that during habituation rats previously treated by MemM walked a longer distance than the other rats, except for the rats previously treated with MemH (*P* < 0.001). Furthermore, on D21 linearity was dependent on condition [*F*_(3, 216)_ = 3.62; *P* < 0.01] (Figure 5B). All rats walked a straighter path (higher linearity values) during tr2 than the ts (*P* < 0.04), while the values were equivalent during tr1 and the ts.

## Discussion

The presented study focused on comparisons of on-going cognitive and non-cognitive processes in the spatial working memory test, the APAAT task, using a wide range of doses of memantine and MK-801. We have shown that a low dose of memantine was associated with a short-lasting improvement in spatial working memory in comparison to the controls. Such an effect was not observed after application of a low dose of MK-801. However, at the low dose no differences in working memory were found between the two drug groups. CSL developed during the first training interval for groups with low doses of the agents. In contrast, high doses of the agents impaired working memory, negatively affected CSL and involved hyperactivity. In the same rats the after-effects of both agents were studied on D21 in the APAAT without drug application. Although all rats performed well in the working memory task and both maintained and updated their skill appropriately according to the novel sector location and the established session conditions, the MK-801H group avoided better during training 2 than the control, MK-801L and MemH groups. In contrast, the non-cognitive indices of locomotor activity (path length and linearity) showed dependence on the training conditions only.

In our working memory variant of the place avoidance test, in order to achieve accurate daily place avoidance, both stimuli segregation and short-term memory was required. The latter being necessary for the formation of new representations of the novel location of the shock sector. Memory acquisition occurred during tr1 and tr2, when the shock was presented, and was tested during the retrieval test (shock inactivated), which started 5 min after the end of tr2. With exposure to these conditions, intact rats improved place avoidance throughout the training sessions, when shocks were presented, and they generally continued to avoid even when the shock was inactivated during the retrieval test (Dockery and Wesierska, 2010). This variant differed from a previously presented working memory variant of place avoidance, wherein the location of the shock sector was alternated from day to day but the daily session did not include a habituation condition (5 min ha before avoidance training in our procedure) and consisted of only a single training interval (Cimadevilla et al., 2000). In such trials, comparison of working memory and CSL within a session was not possible as within a session the two abilities were mutually exclusive. This is due to the fact that in non-alternative variant of place avoidance the rats are always confronted with the same shock-sector location, meaning that they only have to learn the place it occurs in the room-frame coordinates itself. It does not necessitate, however, the application of the rule of how to avoid a to-be-a-frame (in any location), as is required in the alternative variant.

When taking into consideration the three components which comprise the working memory system in animals: goal maintenance, interference control, and memory capacity (Dudchenko et al., 2012), the working memory variant of active place avoidance used in this study seems to be a very useful tool to control the relation between these components. Furthermore, it offers the possibility to test non-cognitive behavior in intact and pharmacologically treated animals. To break down the components according to the APAA task conditions, the place avoidance during tr1, tr2 can be related to goal maintenance, as the rats must use the representation of the shock sector in order to avoid, for a long period of time, entering the actual place where shocks could be delivered. Moreover, interference control also relates to formation of a new representation, which occurs in the consequent session (e.g., from D1 to D2). Thus, interference control would be responsible for proper avoidance in each consecutive session. Better place avoidance performance in tr2 compared to tr1 depends on more effective on-going memory and, in this way, could be related to memory capacity. All components, maintenance, interference control and memory capacity were impaired under NMDARs blockade by high doses, in contrast to low and middle doses of the antagonists, memantine and MK-801. Hence, although the APAAT currently has no complementary human set-up, it has been shown to be a useful tool to study the relation between components of working memory and the underlying mechanisms which control this system.

Working or short-term memory has been previously monitored by other authors/research groups in several animal models of spatial working memory under different doses of memantine or MK-801 with the primary aim to utilize these NMDAR antagonists as therapeutic treatment against excitotoxicity in neurodegenerative diseases (see Ref. in Introduction). In the presented study, acute application of memantine in a dose of 5 mg/kg preserved or even facilitated spatial working memory functioning in the APAAT. Similar to the control rats, this therapeutic dose resulted in a low number of entrances during the training and test conditions and also involved short-lasting enhancement of memory in tr 2, which manifested as a significantly longer maximum time avoided. For comparisons low doses of memantine (0.3; 0.56 mg/kg), but not a higher dose (1.0 mg/kg), enhanced spatial memory after a 18 h delay in the radial maze task, whereas higher doses (3 and 10 mg/kg) totally abolished choice accuracy in the same test (Wise and Lichtman, 2007). In contrast, enhancement of long-term memory was found after a 24 h delay in the radial arm water maze for the 5 and 7.5 mg/kg, but not the 2.5 and 3.75 mg/kg doses. In the same test the same doses had no effect on short–term memory acquisition and retention which was tested after a 15 min delay (Zoladz et al., 2006). Daily continuous infusion of 20 mg/kg memantine by minipumps did not impair working memory in the radial maze (Zajaczkowski et al., 1996). Contradictory to this data, acute memantine application at a high dose (20 mg/kg) negatively affected working memory in our experimental conditions, which manifested as a higher number of entrances with a short maximum time avoided across days.

Discrepancy in the obtained results, as reflected by the data reported here from various behavioral procedures (dry vs. water maze; appetitive vs. aversive conditions), are the result of differences in the memantine plasma concentration during the experimental session, which are dependent on the dose and means of administration (Zoladz et al., 2006).

In view of the similarities and differences between memantine and MK-801, an effect of the latter drug was also studied in terms of memory functioning. Unlike low dose memantine, the low dose of MK-801 (0.1 mg/kg) had no effect on working memory enhancement. However, alike memantine, the high dose of MK-801 (0.3 mg/kg) abolished working memory across the training and test conditions. These results are consistent with recently published data from the Stuchlik Laboratory (Zemanova et al., 2013) which revealed memory impairment in the APAAT after MK-801 in doses of 0.12 and 0.15 mg/kg in naive rats but not in pre-trained rats. Moreover, in the active place avoidance task, MK-801 in the 0.2, but not the 0.15 mg/kg impaired reacquisition of avoidance in the training conditions, whereas both doses impaired place avoidance performance in a new environment (Stuchlík and Vales, 2005). Our experiment was conducted on naive rats in the same environment with doses lower (0.1 mg/kg) and higher (0.3 mg/kg) than those in the previous studies which further supports the reliability of our results. Effect of MK-801 on working memory depending on the dose also in other tests on spatial memory. In the radial maze task working memory was preserved with the 0.05, 0.01, mg/kg 0.08 and 0.1 mg/kg doses and impaired with 0.12, 0.15 or 0.2 mg/kg of MK-801 (Wozniak et al., 1990; White and Best, 1998; Kretschmer and Fink, 1999). However, acquisition of working memory itself and in a new environment in the radial maze was impaired after a 0.0625 mg/kg dose of MK-801 (Shapiro and O'Connor, 1992). Chronic application of MK-801 at a 0.312 mg/kg daily dose by minipumps not impaired working memory in the radial maze test (Zajaczkowski et al., 1996). Contrary to dry radial maze test a single application of MK-801 at the 0.25, 0.5, 2, and 4 mg/kg doses impaired spatial working memory in the water maze on day 1 but this effect disappeared on day 4 (Whishaw and Auer, 1989). It has also been shown that the effect of different doses of MK-801 on spatial memory performance and locomotor activity depend on the experimental procedure, e.g., the water maze test seems to be more sensitive to MK-801 than the dry open field test (Wegener et al., 2011).

To summarize, MK-801 in low doses (0.05 mg/kg, 0.08 mg/kg), was found to have no effect on working memory in naive rats (Wozniak et al., 1990; Kretschmer and Fink, 1999), whereas pre-training in the working memory task preserved memory function even with the higher dose of MK-801 (0.12 or 0.15 mg/kg) (Zemanova et al., 2013). Thus, taking into account the cited literature, we believe that the 0.1 mg/kg dose of (+)MK-801 is sufficiently low, and that lowering it would likely produce negligible effects on working memory in our APAAT.

The shocks/entrance ratio was a useful tool to measure CSL, by which a high ratio expressed poor learning. During habituation, when shock was never applied, the SH/ENTR ratio expresses a “dummy” ratio. Here it was at a similarly high level for all rats, thus confirming that drugs application and the experimental conditions, such as the arena rotation, which was the same for all rats, did not change the rats' spontaneous activity. On D1 the value of the SH/ENTR ratio in all groups was similar for ha and tr1. On this day the task rules were acquired. For D2-3 in all groups, except the high dose groups, the SH/ENTR ratio was lower during tr2 than during tr1 and ha. It means that the rats with low and mild doses of both drugs successfully acquired the task rules. However, on D2-3, it was only for the rats under high doses of both agents that the ratio during tr1 and tr2 was similar with no improvement. That means that high doses of those agents suppressed CSL.

This is in concert with proper performance of working memory and flexible cognitive skill acquisition after low doses of the agents, and consequently memory impairment and disturbed CSL after the high doses related to the within session conditions and an across session learning effect. In spite of a high number of entrances after high doses of memantine and MK-801, the SH/ENTR ratio was at a similar level across session conditions which was similar to other groups (a non-significant group effect). This suggests that high doses of both agents did not disturb the ability to quickly recognize the shock sector and minimize the shocks by escaping, thereby reducing the total number of shocks obtained per entrance even while the number of entrances itself may have been high. Such an effective escape reaction could be due to the higher locomotor activity after high MK-801 across days and high memantine on D3. The different results for working memory performance and CSL could be explained by the heterogeneous nature of the components which belong to the working memory system (Baddeley, 1992; Dudchenko et al., 2000).

Measures of non-cognitive behavior were indexed by the activity characteristics: path length, and linearity. The path length was longer with the high dose of MK-801 independent of day and session condition. Contrary to the high dose of MK-801, hyperactivity related to the high dose of memantine developed throughout the training sessions. Independent of the doses of both agents, all rats walked more during tr 2 than in the other conditions. This result was in opposite to the findings presented by Zemanova et al. (2013), who showed that dose and day but not condition affect the impact of MK-801 on total distance. In training which focused on a stable shock sector location on arena, locomotor activity also increased with the 0.2 mg/kg, but not the 0.15 or 0.1 mg/kg of MK-801 (Stuchlík and Vales, 2005; Vales et al., 2010). In a swimming task the locomotor activity was dose-dependent and time dependent, in which it was impaired 3 h after MK-801 treatment in the doses: 0.25, 0.5, 1.0, 2.0, and 4.0 mg/kg. This was relevant for four of the consecutive post–treatment days, but not for the fifth day (Whishaw and Auer, 1989). Moreover, MK-801 in the 10 mg/kg dose resulted in catalepsy and doses higher than 10 mg/kg were found to be lethal (Whishaw and Auer, 1989). Memantine in a high dose (20 mg/kg) was reported to produce strong motor disturbance in females rats, which was expressed as hypoactivity 10 min after application and progressed to hyperactivity 90 min after drug administration (Creeley et al., 2006). The male rats in our experiments also presented higher locomotor activity after 20 mg/kg memantine but they were able to solve the APAAT task.

Linearity is a unique measure of motor activity that according to our knowledge is currently only calculated using the place avoidance method. Here, a high linearity score (a straighter path) was observed in high dose MK-801 group, which also showed a significantly longer walk distance compared to other groups. Both a long path length and high linearity were noted in rats from the high dose memantine group on all days of training except the first one.

The after-effect of memantine and MK-801 treatment on cognitive and non-cognitive functions was tested with a new shock sector location on D21, 18 days from the last exposure to the agents. Because this period was over the half-life time for memantine and MK-801 (Morè et al., 2008; Wegener et al., 2011) proper place avoidance by rats from all groups was expected without side effects. The results confirmed a lack of differences in working memory performance between the control rats and the rats previously treated with either low or high doses of the agents. This is in accordance with results in which a high dose of MK-801 (5 mg/kg) and memantine (20 or 40 mg/kg) administered intraperitoneally 8 days before training in the radial maze did not affect acquisition of spatial working memory (Zajaczkowski et al., 2000). However, detailed analysis of working memory performance across session conditions shows that during tr2, rats previously treated with the high MK-801 presented a lower number of entrances with a longer maximum time avoided than the groups previously treated with the high memantine dose, low MK-801 dose and the control rats. All rats on D21 showed a similar level of performance for cognitive skill retention/learning. The results primarily show an effect of the session condition in which locomotor activity and linearity was better (short distance, with a low level of linearity) for all groups of rats during the last 5 min of place avoidance training (tr2) than in the other conditions.

## Conclusion

We found that, independent of dose, memantine and MK-80 have different effects on cognitive and non-cognitive functions. Furthermore, dose-dependent effects were found in which memantine in a low dose involved short-lasting improvement in spatial working memory. MK-801 in a low dose, which was found to produce schizophrenia-like symptoms in an animal model, supported working memory performance at the level of the control rats. Both agents in low doses had no effect on non-cognitive functions.

Severe impairment of working memory and disturbance in locomotor activity were produced by the high dose of MK-801 already on the first training session, whereas similar effects with the high dose of memantine developed over time. Differential results for the intra- and across session effects were found for the cognitive processes, working memory and CSL, which confirms that these components reflect the heterogeneous nature of the working memory system. No delayed (long-term) effects of previous drug treatment, except for high dose of memantine, on either cognitive or non-cognitive functions were observed. After a long break without memantine and MK-801 treatment performance was associated with proper working memory and locomotor activity comparable to that of controls. Interestingly, the high dose of MK-801 resulted in improved cognitive performance 18 days after the last drug administration.

The APAAT method offers a valuable and unique behavioral tool to simultaneously compare cognitive and non-cognitive functions under pharmacological intervention. In this study specifically NMDAr blockade, which can help develop concrete models for the therapeutic treatment of neurodegenerative disorders and diseases related to excitotoxicity, was employed. This expands the value of the APAAT beyond its capacity to measure working memory and CSL (Dockery and Wesierska, 2010).

## Authors contributions

Malgorzata J. Wesierska and Colleen A. Dockery designed the research; Malgorzata J. Wesierska, Colleen A. Dockery, equally contributed to ca. 60% of experimentation; Weronika Duda performed ca. 40% of the experiments; Malgorzata J. Wesierska analyzed and interpreted the data and prepared the manuscript; Colleen A. Dockery also prepared the manuscript and was responsible for corrections of the English.

### Conflict of interest statement

The authors declare that the research was conducted in the absence of any commercial or financial relationships that could be construed as a potential conflict of interest.
